# It takes a village: a realist synthesis of social pediatrics program

**DOI:** 10.1007/s00038-018-1190-7

**Published:** 2018-12-24

**Authors:** Ingrid Tyler, Judith Lynam, Patricia O’Campo, Heather Manson, Meghan Lynch, Behnoosh Dashti, Nicole Turner, Andrea Feller, Elizabeth Lee Ford-Jones, Sue Makin, Christine Loock

**Affiliations:** 1 Fraser Health Authority, Surrey, BC Canada; 2 University of British Columbia, Vancouver, BC Canada; 3 St. Michael’s Hospital, Toronto, ON Canada; 4 Public Health Ontario, Toronto, ON Canada; 5 University of Toronto, Toronto, ON Canada; 6 CCFP Iroquois Ridge Medical Centre, Oakville, ON Canada; 7 RD McMaster Children’s Hospital, Hamilton, ON Canada; 8 FAAP, FACPM Niagara Region, Public Health, Thorold, ON Canada; 9 FRCP(C) Hospital for Sick Children, Toronto, ON Canada; 10 Toronto Public Health (retired), Toronto, ON Canada

**Keywords:** Social pediatrics, Realist methodology, Whole child, Interprofessional practice, Health equity, Community-based practice, Trust, Partnership working, Professional education, Empowerment

## Abstract

**Objectives:**

To better understand how social pediatric initiatives (SPIs) enact equitable, integrated, embedded approaches with high-needs children and families while facilitating proportionate distribution of health resources.

**Methods:**

The realist review method incorporated the following steps: (1) identifying the review question, (2) formulating the initial theory, (3) searching for primary studies, (4) selecting and appraising study quality, (5) synthesizing relevant data and (6) refining the theory.

**Results:**

Our analysis identified four consistent patterns of care that may be effective in social pediatrics: (1) horizontal partnerships based on willingness to share status and power; (2) bridged trust initiated through previously established third party relationships; (3) knowledge support increasing providers’ confidence and skills for engaging community; and (4) increasing vulnerable families’ self-reliance through empowerment strategies.

**Conclusions:**

This research is unique because it focused on “how” outcomes are achieved and offers insight into the knowledge, skills and philosophical orientation clinicians need to effectively deliver care in SPIs. Research insights offer guidance for organizational leaders with a mandate to address child and youth health inequities and may be applicable to other health initiatives.

## Introduction

The International Society of Social Pediatrics and Child Health (ISSOP) defines social pediatrics as “a global, holistic and multidisciplinary approach to child health; it considers the health of the child within the context of their society…, integrating the physical, mental, and social dimensions of child health and development, as well as [health] care, prevention and promotion…” (Spencer et al. 2005).

Social pediatrics considers the needs of the whole child (Ford-Jones et al. 2008; Julien 2004). It has been described as a primary health care “linked in and linked across” with specialist health-care services, providing a spectrum of services beyond the traditional health-care system (Wong et al. 2012). Social pediatrics is an equity-oriented practice and philosophy that seeks to take action on the social determinants, such as income, housing, education and environment, as critical mediators of child and youth health.

Social pediatric initiatives (SPIs) target high-needs populations to facilitate proportionate distribution of health resources and provision of services to meet the needs of children and youth living in poverty who are often at higher risk for developmental delay or poor physical or mental health, who often miss out on routine screening, or do not benefit from diagnostic assessment, treatment and/or early intervention (Wong et al. 2012; Shonkoff et al. 2012; Power et al. 2007). While there are a number of equity-oriented clinical practices, those that are explicitly identified as SPIs operate primarily in ISSOP member countries in Europe, as well as Australia, USA and Canada. SPIs aim to improve health and developmental outcomes through increased reach, early intervention and increased service utilization (Ford-Jones et al. 2008). SPIs achieve their objectives through strategies including decentralized delivery of care in defined socio-geographic areas (i.e., place based) (Wong et al. 2012) close linkage with local communities, and cooperation and collaboration among multiple services and service providers (Kodner and Spreeuwenberg 2002) such as physicians, social workers, lawyers and other professionals to foster access to needed care (Julien 2004; Lynam et al. 2010).

Our interest, and the focus of this paper, was to take a realist approach to the examination of SPIs. To our knowledge, there are no systematic reviews of SPIs currently in the literature. Research documenting the need for SPIs spans a broad range, such as the mapping of child developmental vulnerabilities (Canada, Australia, UK), demonstrating the impact of toxic stress on children’s neural development (Brody et al. 2017; Hughes et al. 2017; Luby et al. 2017), and understanding conditions of children facing multiple forms of adversity (Werner 1992). However, there is limited outcome research, and while there have been a number of published studies that have applied the realist review method to complex social and health interventions, there are none that appear to reflect SPIs (Greenhalgh et al. 2007; Kane et al. 2010; Molnar et al. 2015; Pearson et al. 2015; McCormack et al. 2013).

Realist science seeks to generate explanations through observing patterns in the data that recur often enough to support hypothesized mechanisms of action (Pawson and Tilley 1977). These explanations are articulated as CMO (context-mechanism-outcome) configurations. Mechanisms theorize why an outcome was achieved (or not) based on participant reasoning or reaction (Jagosh et al. 2011). Mechanisms are not the intentional strategies or activities of program implementers. Astbury and Leeuw (2010) characterize mechanisms as: (1) “…inferred from patterns in observed behaviour; (2) …sensitive to variations in context … [and] not always be deployed or ‘fired’; and (3) …once ‘fired’ will lead to a specific outcome.”

Realist synthesis involves iteratively building an explanatory framework examining the causal relationships behind an intervention (Jagosh et al. 2013), based on the available literature. The main principle underlying the realist approach is the development and testing of propositions that tie context, mechanism and outcome (CMO) together (Pawson and Tilley 1977). The aim of this realist synthesis is to better understand how SPIs work, for whom and under what circumstances, in order to produce an explanatory model to assist decision makers in developing and planning equitable, integrated, embedded approaches to child and family health.

## Methods

As per Molnar et al. (2015) and in accordance with Pawson et al. (2004), the realist review method we employed is outlined as follows: (1) identifying the review question, (2) formulating the initial theory, (3) searching for primary studies, (4) selecting and appraising study quality, (5) extracting, analyzing and synthesizing relevant data and (6) refining the theory. An advisory committee was selected to incorporate additional knowledge user perspectives into our work. This committee included key stakeholders such as clinicians, researchers, policy makers, public health nurses and representatives of professional organizations. This advisory committee was separate from the research team which included researchers, knowledge users, including pediatricians with expertise in social pediatrics, as well as project staff.

1. Identifying review question
  We derived the review question through an iterative, consultative process with the research team and advisory committee.
2. Formulating initial theory
  We conducted an exploratory literature search and consulted key informants, some of whom were advisory committee or research team members. The exploratory search included terms “social pediatrics,” “community services” and “primary care/public health integration.” Key informants participated in semi-structured interviews describing their experience of SPI programs.
  Our initial theory linked our identified SPI characteristics (C), including (1) an approach concerned with fostering health equity; (2) inter-professional integration; and (3) community embeddedness; to our refined outcomes (O) of interest: (1) improved communication between families and providers, (2) enhanced provider partnerships, (3) increased reach to high-risk families, (4) early identification of health and social risks, (5) increased referral to health and community services and increased service utilization by families; through the following hypothesized mechanisms (M): (1) provider respect, empathy and compassion for vulnerable families; (2) shared values (including health equity and social justice) between providers; (3) community, family and individual trust in providers overcoming hesitancy to engage with the health system; (4) increased trust between providers; and (5) empowerment of families to utilize services.
3. Searching primary literature
  We consulted a health sciences librarian in the development of the search proper, which was informed by our exploratory review described above. The search was started in August 2014 and included all relevant terms related to the following four concepts: children/families, integration, patient care and community services. Health equity terms were covered under the community services concept. A gray literature review was also conducted and supported by a health sciences librarian. These search terms included those from the overall database strategy plus: “social pediatrics,” “child-health whole-child,” and “child-health medical-home.” In February 2015, incorporating ongoing feedback from the advisory committee, the initial search was re-run, recombining concept fields that emphasized our emergent understanding of the relational nature of SPIs. This search also verified that the research team did not miss any important outcome articles. Relevant articles from all sources were hand-searched for additional publications describing SPI programs. Finally, in keeping with a methodological amendment proposed by Jagosh et al. (2011), authors of included papers were contacted to inquire if we had missed any publications or gray literature pertaining to the included SPIs.
4. Selecting studies and appraising the literature
  The inclusion and exclusion criteria for the initial search were developed to test our initial theory and propositions. We included studies pertaining to children and their families from developed countries. This was to maintain some similarity in context among our sample of articles. We excluded interventions that targeted specific age groups or were conducted in low- or middle-income countries (World Bank 2012). We included interventions that were health-care focussed, described how SPIs are enacted at the point of care, and incorporated our three dimensions of social pediatrics. Only studies that reported either qualitative or quantitative outcomes related to our initial theory were included. Through this process, we excluded papers describing implementation of broad social policies such as “Every Child Matters” in England (Bachmann et al. 2009) and “Families First” in Australia (Valentine et al. 2006). Articles describing SPIs but not indicating any measures of success or failure were excluded. All abstracts were reviewed by two research team members, and discrepancies were resolved by consensus.
  Two research team members independently appraised each article based on the following criteria: relevance and rigor (Pawson et al. 2004; McMohan and Ward 2012), depth of description (Arai et al. 2007; O’Campo et al. 2011), and overall validity and reliability (Rosella et al. 2015). A scoring system was created to allow for systematic application of quality criteria; however, articles were not excluded from the review based on a poor quality rating. As described by Pawson (2006), “an otherwise mediocre study can indeed produce pearls of explanatory wisdom.” Instead, our quality appraisal was used to mediate between studies of variable quality, but comparable relevance (Kastner et al. 2011).
5. Data extraction and analysis
  Data extraction was conducted by research team members using a standard form created for this study to record program activities, reported outcomes, successes and failures, and key contextual factors. The purpose of this descriptive data extraction was to generate deep understanding of included articles. All articles related to a specific SPI were grouped together as a “family of articles” in the extraction template.
  The sections of texts from our included studies formed the raw materials for our analysis. All papers were read, re-read and discussed. Throughout this analytic process, the research team met on a regular basis to discuss findings and resolve discrepancies and exchange insights. From our descriptive extraction, we generated explanatory “CMOs” specific to each SPI through a process of retroduction and abduction. Retroduction has been described as “logic of inference,” and its main objective “is to link the structures and causal powers of the subjects under study to the events we want to explain through the notion of causal mechanisms” (Zachariadis et al. 2013). Abductive reasoning involves an iterative process of examining evidence and developing hunches or ideas about the causal factors linked to that evidence. Abduction can be described as “considering all possible theoretical explanations for the data, framing hypotheses for each possible explanation, checking them empirically by examining data and pursuing the most plausible explanation” (Oliver 2012).
6. Theory refinement
  Through individual reflection, partner work, and research team discussions, our analyses of each article were examined according to how it supported, modified, or refuted our initial theory and propositions. In this way, we identified and iteratively refined “demi-regularities,” or semi-predictable patterns where outcomes were linked to context through mechanisms (Molnar et al. 2015).

## Results

### Search and study characteristics

**Fig. 1:**
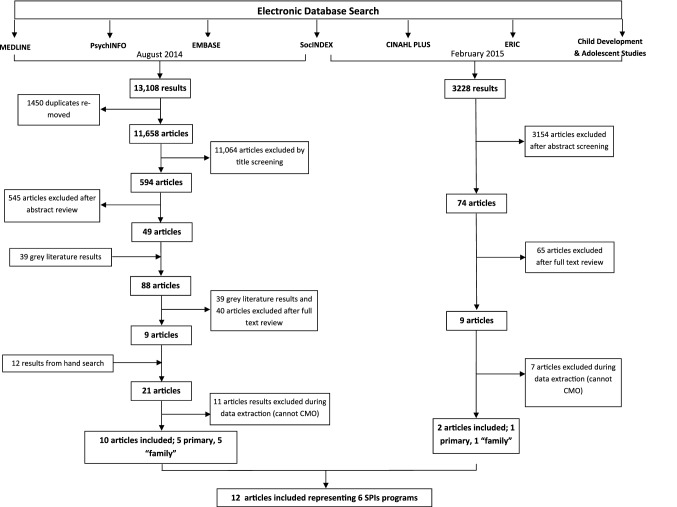
Flow diagram of search, screening and inclusion of articles *CMO* context-mechanism-outcome, *SPI* social pediatrics initiative

As seen in Fig. 1, the search criteria resulted in 14,986 unique and potentially eligible articles. The majority of these articles were excluded on title and abstract review. One hundred and twenty-two articles were identified for full text review, 114 of which were excluded after independent review by 2 research team members. The primary reasons for exclusion were lack of objective outcome data, thin reporting that precluded CMO development or not meeting all 3 identified features that distinguish SPIs from typical clinical services (equity, integration and embeddedness). Table 1 outlines the outcome data which range from validated scales, to program measures, to qualitative analyses. Articles lacking objective outcome data were those that were single author opinion-based assessments of how a program was functioning, or that included program descriptions with no evaluative measures were excluded. Twelve articles were included in the final realist synthesis representing 6 unique SPIs. Characteristics of the included SPIs and related articles are summarized in Table 1.

**Table 1:** Summary of included Social Pediatric Initiatives (SPI), demonstrating health equity, provider integration and community embeddedness, including SPI family of articles, activities and reported outcomes

Program name	Author, year	Study type/ Method	Location	Population/Practitioner	Activities	Outcomes
Early explorers	Barlow and Coe (2013)	Qualitative; semi-structured interviews	Outpatient baby clinics, England	Low-income families (children under the age of 5)/health practitioner and ^b^ECP	^b^ECPs engaging parents in common clinic play area allowing for the opportunity to identify vulnerable families that required referrals	Enhanced service provided within traditional child health clinics (qualitative report) Increased access to hard-to-reach patients (qualitative report) Increase access to services (qualitative report)
	Coe and Barlow (2010)	Descriptive				
Keeping Infants Nourished and Developing (KIND)	Beck et al. (2014)	Quantitative/ time series analysis and descriptive statistics	Hospital medical center, USA	Food-insecure families with infants attending clinic/pediatricians, pediatric residents, and medical students	Collaboration linking food-insecure families to supplementary infant formula, education materials, clinic and community resources or public benefit programs	Increased lead test and developmental screen Increased referrals to social work or medical legal partnership Increase well-baby visits
	Burkhardt et al. (2012)	Quantitative; chart review				Increased identification rate of food insecurity
DentCare	Diamond et al. (2003)	Process evaluation; interview and observation	Harlem and Washington Heights Neighbourhoods, USA	Children in low-income neighborhoods/Columbia University’s School of Oral and Dental Surgery	Provided preventive dental services in schools through collaboration of medical clinics and community-based organizations	Identify major modifications to program required to raise community service to the same priority as education Need for different implementation strategies in different communities Collaboration with community clinics for community linkage
	Albert et al. (2005)	Descriptive				
WE CARE	Garg et al. (2007)	Quantitative; randomized control trial	Outpatient clinic, USA	Low-income families (2 months to 10 years)/pediatric residents	Patient self-administered screening tool and provider community resource book	Greater number of psychosocial issues discussed Received more referrals Greater likelihood of contacting a community resource
Responsive, Interdisciplinary Child-Community Health Education and Research (RICHER) initiative	Wong et al. (2012)	Mixed; Patient interview and survey	Downtown Eastside neighbourhood, Canada	Residents of one of Canada’s lowest income areas/health-care providers	Interdisciplinary collaboration to facilitate access to programs that affect ^a^SDOH	Provider interpersonal style associated with parent reported empowerment scores
	Lynam et al. (2012)	Qualitative; participant observations				Recommendations on fostering engagement and use of indigenous knowledge
	Lynam et al. (2010)	Qualitative; interviews				Illustrate interdisciplinary partnerships enabling clinicians to provide supports to address ^a^SDOH
	Lynam et al. (2011)	Descriptive				
Early Childhood Oral Health Program	Maher et al. (2012)	Evaluation; document review, surveys, interviews	Australia	Infants, young children and their parents/child health professionals	Shared responsibility for oral health, involving a partnership between child health professionals, oral health professionals, and parents of young children	Models of shared responsibility between parents, health professionals and oral health professionals can facilitate primary prevention (routine incorporation of oral health promotion and early identification)

^a^SDOH social determinants of health

^b^ECP early childhood provider

### Demi-regularities

**Fig. 2:**
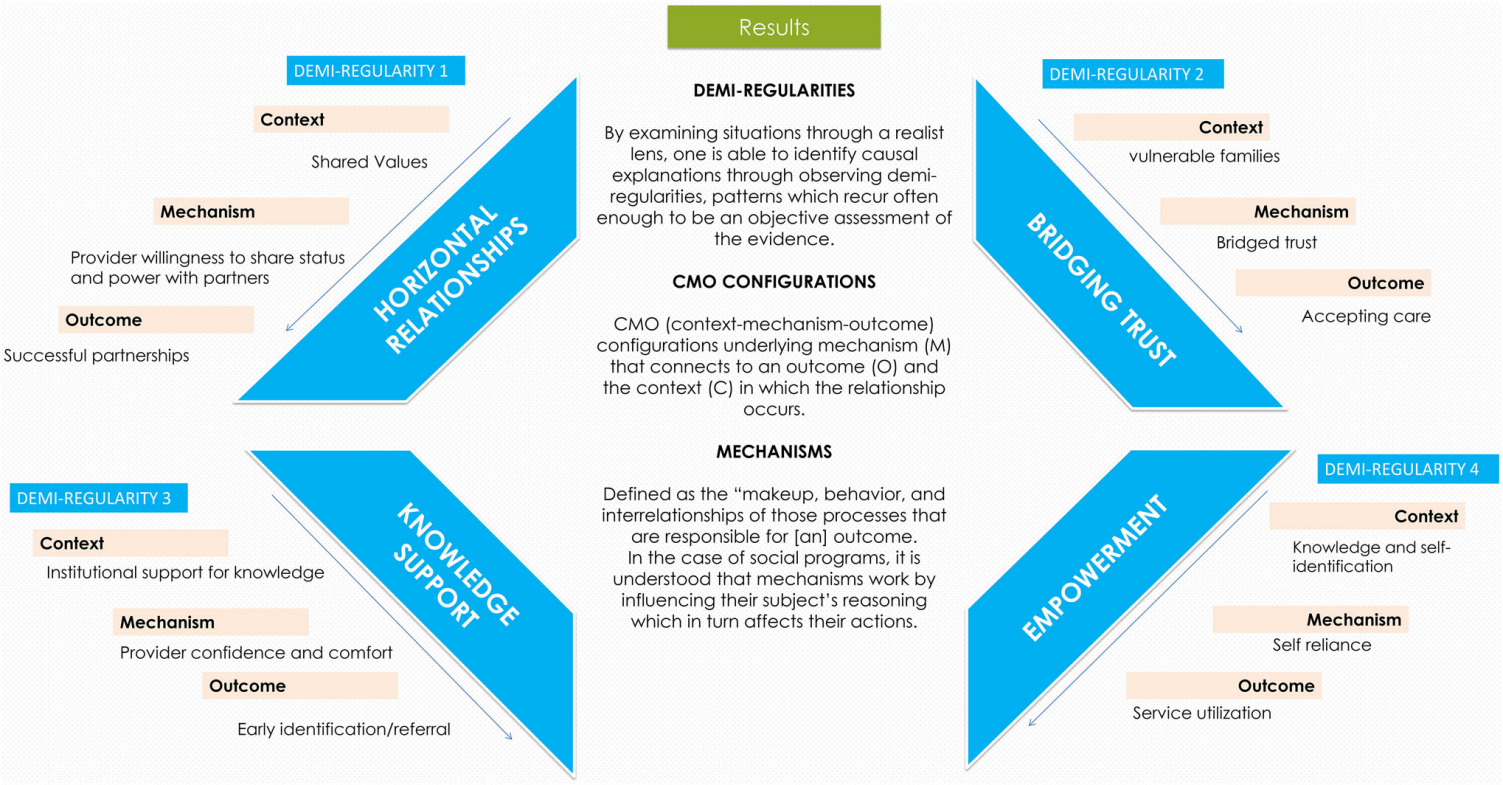
Configurations of four identified demi-regularities, emphasizing thematic results

“Demi-regularities” are semi-predictable patterns where outcomes are linked to context through mechanisms (Molnar et al. 2015). We also noted the specific activities (a) of the program through which outcomes were meant to be enacted. By specifying activities, we were able to separate these from contextual influences. Our analysis identified 4 consistent demi-regularities tying context, mechanism and outcome together and providing explanatory insight into how SPIs may achieve their outcomes (Fig. 2).

#### Demi-regularity #1: Shared values → Willingness of partners to share status and power → Horizontal partnerships

We observed that shared values (C) were a prerequisite for supporting effective partnerships. While our initial theory specified shared values between providers may underlie enhanced provider–provider partnerships (O), the evidence spoke about shared values between organizations. As described by one SPI (Diamond et al. 2003), “Partnership with the community health center worked well because institutional goals were closely aligned.” This contrasted with other failed partnerships previously attempted by the same program that were described as being with institutions that were focused on financial gains, as opposed to increasing services for vulnerable children. Another SPI (Lynam et al. 2010) reporting successful partnerships noted that “[i]t became evident that there was (1) a shared goal (supporting children to achieve their potential), (2) recognition of the different talents each person or organisation brings to the table to achieve this goal; and (3) a commitment to work together.” We found an explanatory mechanism (M) in that an SPI (Barlow and Coe 2013) observed that partnerships were *not* successful because “… a full partnership model would involve… the devolution of status and power to enable the two groups of practitioners with their distinctive but overlapping skills to work more effectively together.” This informed our understanding of SPIs as built upon a structure of horizontal partnerships in which knowledge and power is shared by practitioners.

In addition, the need for institutional support for relationship-building between partners was a common theme. Barlow and Coe (2013) observed a “considerable scope for the further development of these clinics if true ‘partnership’ is to be achieved. This would involve the development of shared aims and objectives, and more extensive and regular training to develop shared agendas, goals and philosophies.” Lynam et al. (2010) pushed the concept of institutional support further and reflected on the need for societal support to achieve the broader SPI goal of health equity: “[o]ur research suggests that if we are committed to fostering access and to reducing inequities, then, the social organisation of systems must reflect the (expressed) commitment to equity.”

#### Demiregularity #2: Vulnerable families → “Bridging” trust → Accepting care

We initially proposed that community engagement and outreach increases family trust in providers and thus increases the reach of the SPI. However, our evidence found that the role of a third party in developing trust is prominent in providing services to disadvantaged, vulnerable or hard-to-reach families (C). Borrowing from social network analysis, we identified that “bridgers,” or critical connecters in a network, enabled trust between the provider and family (Valente and Fujimoto 2010; Issacs et al. 2013), and “bridging trust” (M) is a mechanism supporting increased reach (O).

There was no one single bridging activity that built the community’s trust in providers. In one SPI, the provider’s longstanding relationship with the community was deemed “a key asset drawn upon in building relationships” (Lynam et al. 2010). Another SPI identified that parent–teacher associations are important in one school, whereas an existing community organization proved beneficial elsewhere, and a working group of community leaders was most effective in yet another community (Diamond et al. 2003). This SPI reported that the endorsement of a widely known and respected community leader (a) was effective at “bridging trust” (M) in a “truly disadvantaged community” (C), to overcome a deep suspicion of outside agencies due to a long history of racism and exploitation.

None of the articles reviewed in our study reported increased reach as an objective count of clients seen. Instead, in the context of vulnerable families we conceptualized reach as overcoming families’ distrust and accepting care. In one SPI the development of “bridging trust” occurred through placement of early childhood practitioners (ECPs) in physician waiting rooms to engage parents. These ECPs were seen as more accessible than the physician, and the ECPs were able to gain the trust of hard-to-reach families that visited the clinic, and were described by mothers as being “non-threatening” (Barlow and Coe 2013). This is congruent with the finding of another SPI reporting that, “…the provider’s interpersonal style of compassion and respectfulness … likely improves the provider’s and patient’s perceptions of trust in each other” (Wong et al. 2012).

#### Demiregularity #3: Institutional knowledge support → Practitioner confidence → Increased number of client referrals to needed services

We initially proposed that providers must be comfortable in identifying social risks, such as low income, with respect, empathy and compassion. We also proposed that practitioner confidence in other service providers would increase referral to health and community services. We found both of these propositions supported by the literature and linked to institutional support of knowledge translation and partnership building. With institutional support (C), providers were better able to identify risk and make community referrals (O) through increased comfort and confidence (M).

As SPIs are equity-focussed initiatives, substantial research (Commission on Social Determinants of Health 2008; Margolis et al. 2001; Ministry of Health (BC) 2007) and our own practice experiences confirm that typical clinical practices are not organized to address SDOH. The literature reviewed underscored this point and provided examples of ways SPIs sought to fill this gap. For example, in Garg et al. (2007) pediatric residents received training about community resources, as well as instruction on screening families’ psychosocial problems. Residents who received the training described feeling more comfortable when screening and more confident to refer to the community resources. Parents in the intervention group had significantly greater odds of receiving referrals to community resources. However, the authors stated that “relatively few referrals were made for sensitive topics suggesting residents may be less comfortable with these subjects and may require further training.

Another SPI incorporated education about food insecurity and its effects. Specifically, “providers were introduced to the program [through] educational sessions on food insecurity… [and] received a tour and onsite training at the food bank” (Beck et al. 2014). Identification of food insecurity rate increased from 1.9 to 11.2% during the intervention period. Provider tools, such as EMRs [electronic medical records] and questionnaires, “…empower[ed] both providers and families to feel comfortable discussing sensitive topics” (Burkhart et al. 2012).

Lastly, Maher et al. (2012) identified “lack of knowledge about oral health, not feeling confident to deliver oral health messages and feeling it may cross professional boundaries to do so” as a significant access barrier. They initiated accessible training across interdisciplinary providers leading to an increase in referrals to public oral health services.

#### Demiregularity #4: Vulnerable families → Self-reliance → Service utilization and referral follow up

Although we initially proposed empowerment as a mechanism that would increase service utilization by families to meet a variety of their health and social needs, our analysis identified that the term “empowerment” was being presented differently across studies and that it was best conceptualized as an activity or process that increased client knowledge or supported clinician empowering behaviors.

The mechanism identified as underlying the various conceptualizations of empowerment is client self-reliance (M). In the context of marginalized populations (C), these empowerment activities “influence, shape and increase parental confidence and ability” (Barlow and Coe 2013) or “invoke a sense of … mutual responsibility between parents and providers…” (Garg et al. 2007). Where empowerment was measured as an outcome of respectful engagement with practitioners, clients realized the value of their existing expert knowledge and skill not only for themselves but also for their families and communities (Lynam et al. 2012).

In Garg et al. (2007), an intervention group empowered to self-identify problems and to indicate their motivation to address them through a self-report survey tool had a significantly higher rate of discussed psychosocial topics compared to the control group. At 1 month, 20.0% of the parents in the intervention group reported contacting a referred community resource versus 2.2% of parents in the control group. Overall, 34% of referred intervention parents reported contacting a community resource (O).

Wong et al. (2012) sought to empower parents by guiding them to acquire knowledge of their child’s health condition or developmental stage, offering management strategies and connecting them with resources. Clients were empowered to manage their health and the health of their families because they learned how to navigate the health-care system and use the network of supportive services operated by community agencies.

Lastly, Beck et al. (2014) and Burkhardt et al. (2012) provided infant formula supplements to food-insecure families and sought to empower patients to speak up about social concerns through posters in examination rooms that explained the clinical focus on social determinants of health and services available. The authors hypothesized that through interactions related to the provision of infant formula families felt more empowered to return to the clinic for consistent well-care and support for other social challenges.

## Discussion

This realist synthesis aimed to identify processes of care (through CMOs) that may improve health and developmental outcomes for children and youth coping with adverse social and material circumstances. Our analysis identified four consistent patterns tying together contexts (shared values, vulnerable families, institutional support), mechanism (sharing power, self-reliance, bridging trust, practitioner confidence) and measured outcomes, such as successful provider partnerships, increased client reach, and increased referral to health and community services to provide explanatory insight into how SPIs may achieve their goals. The study is unique because it has focused on the process of “how” outcomes are achieved (i.e., mechanisms), thereby offering direction for the knowledge, skills, and philosophical orientation clinicians need to effectively develop relationships and form partnerships in SPIs.

Lynam et al. (2011) identify that a central, yet often unexamined, assumption of primary health care is that families have the knowledge, skills and resources to navigate the health-care system, follow through on referrals, enact recommended treatment, and clearly present their concerns about their child to health-care providers in order to initiate treatment. However, in the context of vulnerabilities that arise out of social, material and historical circumstances, many parents are not “confident advocates” for their children, or parents may recognize a need for support for their child but their ability to act on the need is constrained by a number of factors, including a lack of knowledge of child development, health systems organization, or of their rights. In addition, there were indications in our data that practitioner comfort and confidence needs to extend into the area of providing care to vulnerable populations in general. Practitioner knowledge of the social, material and organizational conditions that contribute to inequities appears to be critical to the delivery of appropriate referrals for social and medical conditions. Wong et al. (2012) argue that empowering patients through knowledge allows families on the margins to become more active participants in their care and that perceived empowerment was related to clinician behavior. We found both empowerment processes were linked to increased use of services.

Trust and empowerment are shown to be fundamental cornerstones of successful SPIs. Both were present in different ways for practitioners and patients. Practitioners needed to trust each other, and they needed to be empowered by their organizations to pursue this “different kind of care,” a phrase coined by Julien (2004). Patients needed to overcome barriers to trust their practitioners in order to be empowered by the SPI for treatment of social and medical circumstances. Notably, we found that the context and history of the target population likely had a significant effect on the way the programs worked to build trust which influenced their successful delivery.

Lastly, our understanding of SPIs is that they are built upon a structure of horizontal partnerships in which knowledge and power is shared by practitioners, partners and families. Our conceptualization of horizontal partnerships encompasses the principle of symbiosis noted in horizontal collaboration (Inside Supply Management 2012) and the concept of limiting hierarchy noted in horizontal business organization (Quain 2018). It also includes horizontal communication, which legitimizes and validates community-based knowledge, derived from lived experience and local conditions (Bradford 2005). This is in keeping with analyses of primary health-care quality indicators and feminist theorizing about conditions that foster the broadest forms of engagement across individuals and organizations with differential power, in part because it recognizes and values different forms of expertise. In addition to the critical point of sharing power with other service partners, authors currently practicing with SPIs describe working environments embodying horizontal partnership where clients are considered an equal partner, where power is shared across patients and providers, where leadership is devolved, and group decisions and actions are driven almost exclusively by the shared vision of improving outcomes for vulnerable children and families.

A strength of this research was the close involvement of practice-based knowledge users. Limitations stem from a lack of clear definition of the social pediatrics term and a paucity of current literature exploring social pediatrics as a defined practice model. For this realist review, we focused on clinical programs that operationalizing all 3 dimensions of the social pediatrics philosophy (equity, integration and embeddedness). We excluded broad social policies that also met these criteria but did not have comparable outcomes for the purposes of this realist review. In addition, issues of attribution would arise in looking at these policy evaluations (for which there was also limited literature available). While we did find published descriptions of SPIs, there were limited evaluations reporting measured qualitative or quantitative outcomes available for comparison. As a result of the available literature, our results were highly influenced by one SPI in particular, the Responsive Interdisciplinary Child-Community Health Education and Research (RICHER) [4], which had the largest number of related articles that also provided the thickest descriptions of context and mechanisms.

This paper contributes to a limited evidence base on delivery of the SPI philosophy of care. The next steps in the research process are to identify the attributes of practitioners and clinicians who engage in inter-sectoral work. We would also like to discern the organizational features, such as policies and resources, which are needed to successfully introduce the suggested model of practice. Finally, future research needs to be done to identify the range and nature of the outcomes that can be achieved through an integrated, embedded, and healthy equity focused social pediatrics approach. By identifying the organizational structures that underpin integrated outreach partnership models of care, this research insights offer guidance for organizational leaders whose institutions/programs have a mandate to address child/youth health inequities and may be applicable to other health services initiatives aiming to reduce inequities.

## Abbreviations

CMO: Context-mechanism-outcome
ECP: Early childhood practitioner
EMR: Electronic medical record
ISSOP: International society of social pediatrics and child health
RICHER: Responsive interdisciplinary child-community health education and research
SPI: Social pediatrics initiative
